# The role of visual experience in haptic spatial perception: evidence from early blind, late blind, and sighted individuals

**DOI:** 10.1186/s13293-025-00747-y

**Published:** 2025-08-19

**Authors:** Lara A. Coelho, Daniela E. Aguilar Ramirez, Serena Basta, Marta Guarischi, Claudia L. R. Gonzalez, Monica Gori

**Affiliations:** 1 Unit for Visually Impaired People (U-VIP), Italian Institute of Technology, Genoa, Italy; 2 The Brain in Action Laboratory, University of Lethbridge, Lethbridge, AB Canada; 3 Department of Informatics, Bioengineering, Robotics and Systems Engineering (DIBRIS), University of Genova, Genoa, Italy

## Abstract

There is contradictory evidence on the effect that visual experience has on haptic abilities. Indeed, some studies have documented that a lack of vision (blindness) results in decreased haptic perception, whereas other studies report an enhanced haptic ability in blind individuals. To examine the role of vision in haptic spatial processing, we recruited early blind, late blind, and sighted participants. Each participant completed a haptic task in which they explored a two-piece LEGO model for eight seconds before searching for the same pieces in a bowl of distractors. Our results showed that blind individuals made more errors than sighted participants. Furthermore, early blind participants performed worse than both late blind and sighted participants, who performed similarly. These findings highlight the importance that vision plays in the development of accurate haptic spatial perception. Additionally, we investigated whether the commonly reported male advantage in haptic tasks depends on visual experience. Our results showed better performance by males in all groups when compared to females. This result suggests that sex differences in haptic spatial processing are a fundamental characteristic of human sensory function, independent of visual experience.

**Highlights**

- No study has investigated if the previously identified male advantage in haptic spatial processing is mediated by visual experience.
- Blind participants made more errors than sighted participants; early blind performed the worst.
- The findings suggest vision is crucial for the development of accurate haptic spatial perception.
- There was a consistent male advantage in haptic performance across all visual experience groups.
- Sex differences in haptic spatial ability appear to be independent of visual expertise.

**Plain language summary**

Some researchers have suggested that being blind reduces abilities in their other senses, while others believe that a lack of vision can improve them. To further understand which is true, we investigated whether the haptic system—the combination of touch and proprioception (awareness of where the body is in space)—is affected by blindness. To do this, we tested people who were blind from birth (early blind), people who became blind later in life (late blind), and people who can see (sighted) on a simple haptic task. In the task, participants felt a small LEGO model with their hands for eight seconds. Then, they had to find the same LEGO pieces in a bowl filled with other, distractor pieces—using only haptics. We found that blind participants made more mistakes than sighted participants. Those who were blind from birth had the most difficulty. People who became blind later in life performed similarly to sighted individuals. This suggests that vision plays an important role in developing accurate haptic perception. As previous work has shown that males outperform females on haptic tasks, we also investigated whether those differences depended on vision. We found that males performed better than females in all groups, regardless of whether they were blind or sighted. This suggests that sex differences in haptic ability may be a basic feature of how our senses work and not just related to vision.

## Introduction

During our daily lives, we receive stimuli to our various sensory modalities (e.g., vision, touch, proprioception, audition). When we consolidate multiple sensory modalities into a single percept, this is a process called multisensory integration. While the integration of these stimuli is critical, specific senses play a more prominent role for certain cognitive abilities. For example, in spatial cognition vision has a pivotal role for humans [4, 15, 28, 32]. In fact, previous studies have shown that the visual modality can offer a spatial background for remapping other sensory information [16]. In other words, during development vision “teaches” the other senses relevant cues about space. The importance of early visual experience on spatial abilities has also been reported in non-human primates [35]. Furthermore, multisensory neurons are present in newborns, and they develop their spatial-specific multimodal properties based on early experiences [16, 35].

The importance of visual experience across development is also apparent from results showing that blind adults struggle with a variety of spatial tasks across sensory modalities (e.g., audition, touch, locomotion [4, 17, 19, 26]. For example, when asked to complete an audio spatial bisection task, Gori and colleagues found that early-blind individuals were unable to determine the spatial proximity of the sounds [17]. In that task, participants were seated in front of an array of speakers and were presented with three sounds coming from various speakers. The participant had to determine whether the second sound was closer in space to the first or third sound. The authors found that unlike sighted individuals, the blind participants based their estimations on the temporal rather than the spatial cues relevant to the task. This work, along with others (e.g., [4, 19, 26]) have contributed to the hypothesis that visual experience is necessary to develop an accurate spatial representation across various senses. Intriguingly, in the work of Gori et al. [17], the authors found that blindness onset determined their results. Indeed, they observed that those who lost sight later in life (late-blind individuals) did not always show the same deficit in spatial perception as the participants who had been blind since early childhood (early blind). This aligns with the proposal that early visual experience calibrates spatial representations in the other senses. Therefore, early and late-blind individuals may significantly differ in their spatial abilities.

One sensory modality that blind individuals rely on heavily to interpret their surroundings is haptics. Haptic ability is defined as the combination of tactile and kinesthetic feedback [20]. Blind individuals frequently rely on the haptic sense to understand spatial relationships and form mental representations of space. However, the literature presents mixed findings regarding whether the lack of visual experience enhances or impairs haptic spatial perception. It has long been proposed that losing a sense leads to an improvement in the other remaining senses (e.g., haptics), and some research has shown that to be the case [12, 25, 29, 36]. For example, Norman and Bartholomew [29] found that blind individuals outperformed sighted controls on both a tactile grating orientation and a three-dimensional haptic shape discrimination task. This finding may reflect that blind individuals have more haptic experiences and rely more on haptics to navigate and interact with the world. However, other studies have found that blind individuals have impaired haptic spatial perception [5, 18, 23]. For example, Fiehler et al. [14], compared sighted participants to congenitally blind individuals and found that the blind performed significantly worse on a haptic spatial task than their sighted counterparts, suggesting that early visual experience may be necessary for the optimal development of haptic spatial processing. Similarly, Postma et al. [33] found that early blind participants performed worse than late blind individuals on a haptic task requiring allocentric spatial representations, and that both groups of blind individuals performed worse than sighted participants. One last possibility is that blind and sighted individuals will perform similarly on haptic spatial tasks. There are several studies showing that blindness does not influence performance on haptic tasks [21, 27, 30]. For example, in the study by Overvliet et al. [30] blind and sighted individuals were asked to haptically explore three different objects, two identical and one different, and identify the odd one out. The results showed that blind participants performed similarly to sighted participants in object recognition. The authors concluded that the development of spatial processing in haptic recognition matures relatively late, independent of visual experience. Thus, it remains unclear whether the absence of visual experience impairs, enhances, or has no effect on haptic spatial abilities.

The purpose of this study was to investigate how different visual experiences (early blind, late-blind, and sighted) mediate performance on a haptic spatial task and whether biological sex plays a role. Prior research suggests that males may outperform females on haptic spatial tasks [1, 10, 34]. For example, in a recent study, Aguilar Ramirez and Gonzalez [1] found that male participants were faster and made fewer errors than female participants when using haptics to identify simple objects. However, it remains unclear whether visual experience contributes to these sex differences. In a previous study investigating sex-differences in body representation tasks, it was found that males were more accurate than females regardless of visual ability [8, 9]. This finding suggests that studies investigating haptic ability in visually impaired individuals should consider sex as a variable. We recruited a group of early and late blind adults along with a sighted control group and asked them to perform the same task as in [1]. In this task, participants were presented with a 2-piece LEGO model, and they were asked to haptically explore it for eight seconds (sighted participants wore a blindfold). After the exploration of the model, the two pieces that made up the model were placed inside a bowl containing 10 other unique pieces. Participants were asked to find the two pieces that made up the model as quickly and accurately as possible. They completed this task both with their left and right hands separately. Our first hypothesis is that early blind participants, lacking visual calibration from birth, will make more errors and take longer to complete the task than both late blind and sighted participants. Our second hypothesis is that, due to the male advantage in haptic processing, males will outperform females in the control group. Based on the result of [8, 9] which showed that blind male participants performed better than female blind participants, we predicted sex differences in the current study.

## Method

### Participants

Fifty-two (n = 31 females) sighted healthy right-handed participants between the ages of 18 to 59 years old and sixteen (n = 9 females) early and late blind right-handed participants between the ages of 26 and 59 took part on this study. Participants self-reported their handedness, sex, and gender; all identified as cisgender. All sighted participants were right-handed, while blind participants were either right-handed or reported no hand preference. In total nine late blind and seven early blind individuals participated in this study. All early blind participants reported being blind from birth. The blind participants had a variety of different pathologies and blindness onset. Details related to diagnosis and onset of blindness are summarized in Table 1. Sighted participants were recruited through the Department of Psychology at the University of Lethbridge, using participant management software (Sona Systems). These university students received course credits for their participation. Blind participants were recruited through a database established within the UVIP group at the Italian Institute of Technology. The experiment was approved by the University of Lethbridge Human Subject Research Committee and by the Comitato Etico Regionale della Liguria (Genova, Italy), application number 11174.

**Table 1:** The descriptive information about each of the blind participants

Participant	Sex	Diagnosis	Blindness onset (age in years)	Age	Early/late	Residual vision
1	Female	Nystagmus and retinitis pigmentosa	35	59	Late	Lights and shadows
2	Female	Congenital glaucoma and retinal detachment	0	30	Early	No vision
3	Male	Degenerative oculopathy in both eyes	20	36	Late	Lights and shadows
4	Female	Leber’s congenital amaurosis (LCA)	46	59	Late	No vision
5	Male	Leber’s amaurosis	0	53	Early	No vision
6	Male	Brain surgery	19	29	Late	Lights and shadows
7	Female	Retinopathy of Prematurity	0	35	Early	No vision
8	Female	Retinitis pigmentosa	25	58	Late	No vision
9	Male	Leber’s amaurosis	0	32	Early	No vision
10	Female	Retinopathy prematurity	0	26	Early	Lights and shadows
11	Male	Damage to optic nerve	24	46	Late	Lights and shadows
12	Female	Retinopathy of prematurity	0	38	Early	No vision
13	Male	Corneal opacity	17	37	Late	No vision
14	Female	Loss of retina	18	48	Late	Lights and shadows
15	Female	Retinitis pigmentosa	0	36	Early	Lights and shadows
16	Male	Retinitis pigmentosa	34	49	Late	Lights and shadows

Sex, diagnosis, age of blindness, age at testing, and if they were early/late blind are all reported

### Task

**Fig. 1:**
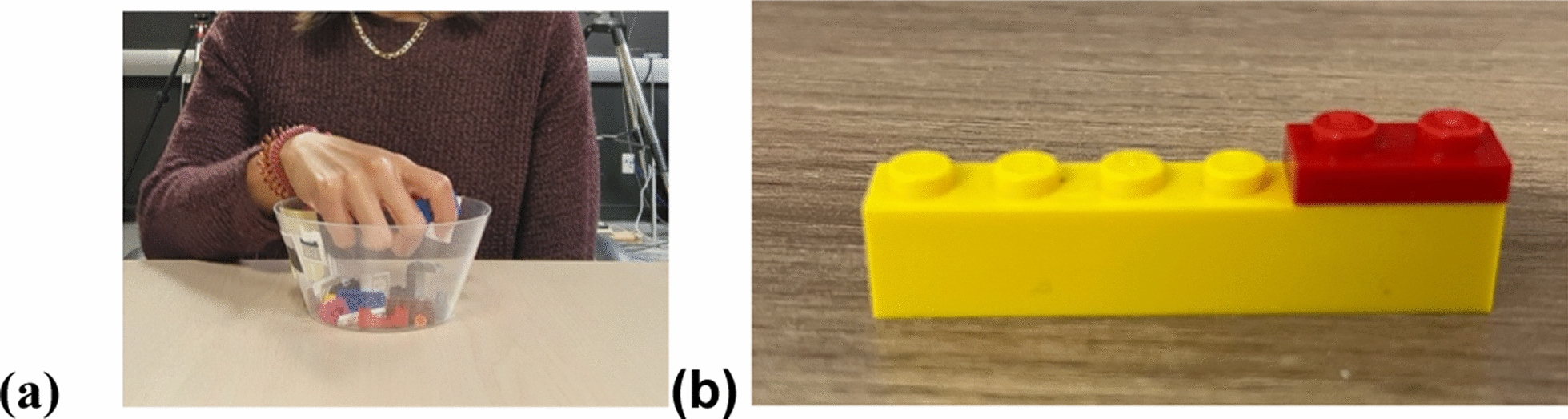
The haptic task. **a** The set up- participant picking from a bowl with 12 distinct LEGO blocks, the two that they thought made up the model. **b** An example of a 2-piece LEGO model

We used the same task and methodology as Aguilar and Gonzalez [1]. This haptic task is illustrated in Fig. 1. The task comprised 12 trials, with six trials performed using the left hand and six with the right hand. The trial sequence was randomized for each participant, and the starting hand (left or right) was counterbalanced across participants. During each trial, blindfolded participants haptically explored a simple two-piece LEGO model (Fig. 1) for eight seconds using one hand. Each of the 12 models was unique, composed of two distinct LEGO pieces. Following the exploration, the two pieces were placed in a bowl containing 10 additional, unique distractor pieces. Participants were then required to haptically search the bowl to identify and retrieve the two LEGO pieces that constituted the model they had just explored. In total, the bowl contained 12 pieces, including the two target pieces and 10 distractors.

### Procedure

Participants initially reviewed and signed a consent form in accordance with the Declaration of Helsinki. Subsequently, they were seated at a table (Fig. 1). Sighted participants were blindfolded to ensure comparable sensory conditions across all participants. They were instructed that they would briefly touch a model composed of two LEGO pieces and that their task was to search for the two specific pieces that formed the model among a set of distractors. Moreover, participants were informed that they were permitted to use only one hand (either left or right) during the task. Once the pieces were identified, participants had to remove them from a bowl and place them on the table beside the bowl. They were encouraged to complete the task both accurately and as quickly as possible. The experimenter provided participants with a blindfold to ensure visual occlusion. A bowl containing 12 LEGO pieces (10 distractors and the 2 target pieces) was positioned on the table in front of the participant. The experimenter then placed the model (composed of two connected pieces) onto the participant’s designated hand, allowing them to touch the model for eight seconds. Timing began when the experimenter placed the model on the participant’s hand and ended when the experimenter indicated that the time had elapsed. Following this, the experimenter disassembled the model, placed its two pieces into the bowl with the distractors, and initiated the stopwatch as the participant began searching for the target pieces within the bowl. The stopwatch was stopped when the participant successfully placed the two target pieces outside of the bowl and onto the table. This procedure was repeated for each trial.

### Data analysis

For the haptic task, there were two dependent variables: number of errors and time to complete the task. An error was recorded if the participant took out of the bowl a piece that did not match any of the two pieces that made up the model. Additionally, the time taken to find the pieces inside each of the bowls (i.e. each trial) was recorded.

We conducted two-way ANOVAs on the dependent variables (errors and time) with group (blind, sighted) and sex (female, male) as between-subjects factors. These analyses addressed our primary question: (1) Does the absence of visual experience affect haptic spatial perception? and our secondary question: (2) Are there sex differences in haptic spatial perception, independent of visual ability?

JASP (version 0.16.4) was used for all analyses. The alpha level for all comparisons was 0.05.

## Results

### Sighted vs. blind

While we acknowledge the discrepancy in group sizes, with a considerably larger control group (N = 52) compared to the blind participants (N = 16), we conducted additional analyses using an age- and sex-matched subset of the control group. These analyses yielded results consistent with those obtained from the full sample. Therefore, we chose to include the entire control group in our final analyses to provide a more representative sample of the sighted population.

*Errors:* There was a main effect of sex [*F*_(1,64)_ = 7.95, *p* < 0.01, η^2^ = 0.09] as males (2.09 ± 0.69) made fewer errors than females (3.51 ± 0.64). There was also a main effect of group [*F*_(1,64)_ = 13.28, *p* < 0.01, η^2^ = 0.16] as blind participants (3.72 ± 1.03) made more errors than sighted participants (1.88 ± 0.31). The interaction between group and sex did not reach significance [*F*_(1,64)_ = 0.002, *p* = 0.97, η^2^ = 0.000002].

**Fig. 2:**
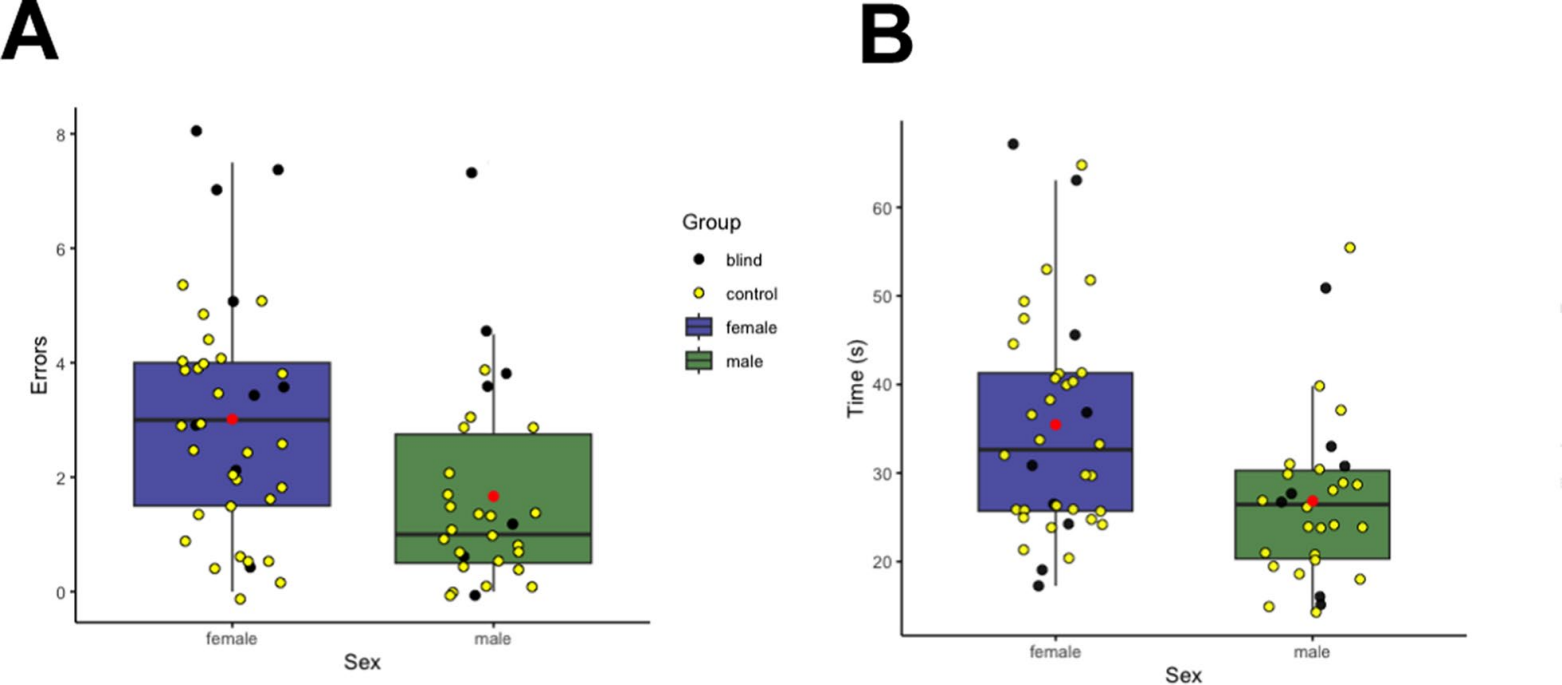
Haptic task errors and times for each sex (female, male)

*Time(s):* There was a main effect of sex [*F *_*(*1,64)_ = 5.51, *p* = 0.02, η^2^ = 0.08]; males (27.62 ± 3.76) were faster at finding the two pieces compared to females (35.64 ± 4.33), see Fig. 2. There were no other main effects or interactions, for summary see Table 2.

**Table 2:** The statistics for the results that did not reach significance when time was the dependent variable

Effect/interaction	*F*	*p*	η^2^
Group	0.373	0.54	0.005
Group × sex	0.0004	0.98	0.00001

Blindness onset is a key factor in understanding how visual deprivation affects sensory processing and cognition [2, 3, 16, 35]. To explore its potential impact on our haptic spatial task, we conducted an exploratory analysis using two-way ANOVAs with blindness group (early vs. late) as a between-subjects factor. Although the sample sizes were small, this analysis offers a useful foundation for future research.

### Exploratory analysis: early vs late blind

**Fig. 3:**
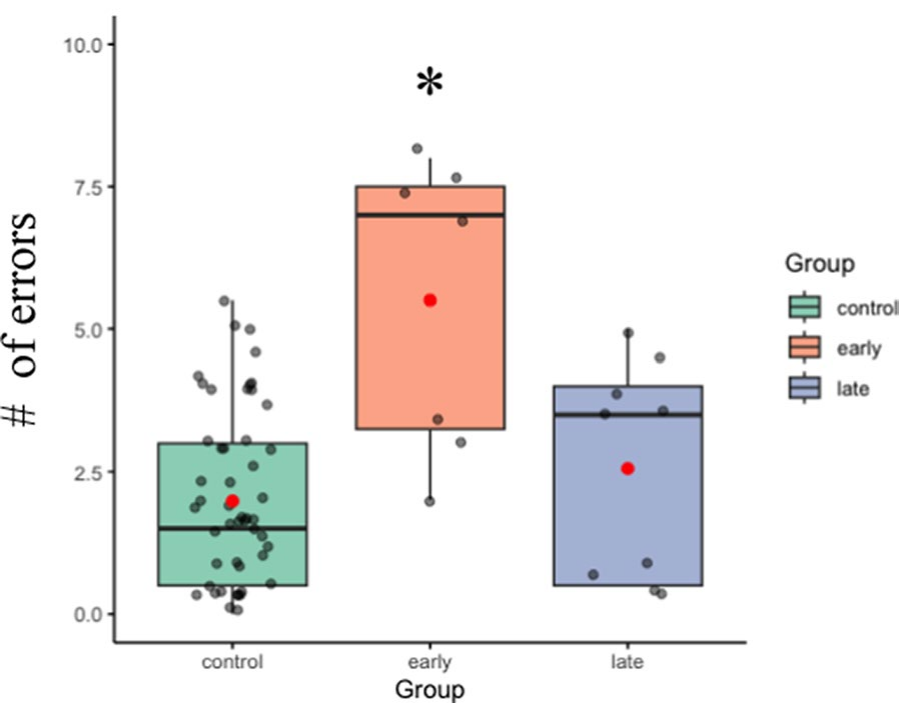
Haptic task errors for each group (control, early and late-blind). Control (*green*) and late-blind participants (*blue*) performed similarly. Early blind (*orange*) made more errors than both the other groups. The *red dot* indicates the mean and the *horizontal black line* in each bar graph is the median. There were no main effects or interactions for the times in the haptic task.

*Errors:* There was a main effect of group [*F*_(2,65) =_12.76, *p* < 0.01, η^2^ = 0.28]. Follow-up Bonferroni corrected paired-samples t-tests revealed that early blind participants (5.5 ± 0.99) made significantly more errors (*p*’s < 0.01) than both late blind (2.5 ± 0.82) and sighted participants (1.99 ± 0.25), see Fig. 3. There was no difference in performance between late blind and sighted participants (*p* = 1).

*Time:* There was no effect of group [*F*_(2,65)_ = 0.16, *p* < 0.85, η^2^ = 0.005]. This suggests that participants did not compensate for making fewer mistakes by going slower (and vice versa).

### Relationship between blindness duration and performance

There was a significant correlation between the percentage of life spent blind and total errors made (*R* = 0.91***, *p* < 0.001). The longer the participant had been blind (in relation to their own age), the more errors they made.

Furthermore, there were no relationships between the time to complete the task, and the number of errors made (*R* = 0.21, *p* = 0.08). This indicates that those who took longer to complete the task did not compensate by making fewer errors (and vice versa). In fact, if anything it was trending in the other direction, those who took longer were making slightly more mistakes.

## Discussion

The present study evaluated if visual experience modulates haptic abilities. To test this, we recruited a group of early and late blind individuals along with a sighted control group and asked them to complete a simple haptic task. In this task, participants haptically explored a two-piece LEGO model for eight seconds and were then asked to identify the two pieces that comprised the model in a bowl containing 10 distractor pieces. We recorded the number of errors made and the time it took the participants to complete the task. Our results showed that blind participants made more errors compared to controls. This was especially true for the early blind individuals who performed significantly worse than both sighted and late blind participants. Moreover, there was a main effect of sex, in which females made more errors and took longer to complete the task compared to males. This was true regardless of visual experience.

The main result of our study is that a lack of visual input decrease’s haptic ability, as we found that blind participants, overall, made more errors haptically compared to sighted controls. These results counter the argument that losing one sense enhances abilities of the remaining senses. Therefore, our results support the hypothesis that vision is needed to calibrate spatial representations of the other senses [4–6, 13, 14]. Indeed, haptic spatial processing has been reported to improve with visual availability [34]. In that study non-informative vision was shown to improve performance on a haptic parallelity task. Those participants were asked to match the position of a bar (hidden from view, to that of a reference bar of which the participant had full vision). They found an increase in performance when vision of the reference bar was allowed. This suggests that vision, even when non-informative, supports haptic processing, highlighting its role in guiding spatial representations. The results from the present study support this idea. With respect to blindness, our findings align with previous works which argue that the integration of touch and proprioception (haptics are affected by a loss of sight. For example, Ehrsson et al. [13] showed that blind individuals are immune to the somatic rubber hand illusion. In this experiment, the participants hand is taken by an experimenter and together they brush a dummy hand, meanwhile simultaneously the experimenter strokes the participants’ own hand. This simultaneous stroking causes a shift in embodiment to the dummy hand in sighted individuals (even though they are blindfolded), however, blind individuals are not affected by this illusion. While stroking the dummy hand, the participant should feel a conflict between proprioceptive (where their hand is in space) and haptic (the information from brushing the dummy hand), with the haptic information causing the participant to merge their own hand and the dummy hand into a unitary percept (causing embodiment of the dummy hand). Those authors argued that blind participants do not have the ability to re-map tactile feedback into an external frame of reference, which is a skill that sighted individuals often report using. We postulate that this may also explain our results. Sighted individuals could remap the haptic feedback about the LEGO pieces into a visual reference frame, which allowed them to make fewer errors. Our results suggest that haptic feedback as a whole—including tactile, proprioceptive, and the cognitive spatial processes involved in forming mental representations—is also impaired in blind individuals.

One possibility is that the sighted individuals used the image-mediation model (i.e., visual imagery) to help guide their performance, a strategy that blind participants could not engage in. The image-mediation model refers to the process of haptic input being translated into a visual image [24, 31]. Previous research has showed that blind individuals struggle in haptic recognition performance because they cannot use the image mediation model [24]. In that study, participants were asked to haptically explore different raised line drawings of familiar objects (e.g., cup, hammer, book) with one or both hands and to identify the shape as quickly and accurately as possible. Sighted participants were also asked to visually rank the vividness of the raised images. The authors found that sighted individuals’ visual responses strongly correlated with their haptic performance, suggesting that visual imagery plays a key role in haptic recognition. In contrast, blind individuals performed significantly worse than sighted participants, and their performance did not correlate with 3D haptic estimation. These findings support the idea that our current results may reflect differences in image-mediated processing.

The results of our exploratory analyses, show that sighted and late blind participants performed better than early blind participants, indicating that visual experience may modulate haptic abilities. Moreover, we found a significant correlation between the number of errors) and the time since blindness onset. These results support previous arguments that early visual experience is necessary to “teach” the other systems about space [16, 35]. Relevant to our findings are a couple studies which have shown that the duration of blindness affects spatial skills [2, 3]. For example, the work of Amadeo et al. [2] demonstrated that the duration of blindness influenced gradual changes in the neural circuits responsible for constructing spatial representations. In that EEG study, participants were asked to perform the same audio-spatial bisection task as in [16]. The authors found that a shorter period of blindness resulted in a better performance on the audio-spatial bisection task and in a greater contralateral activation in the visual cortex, similar to sighted participants. This finding highlights how neural changes in spatial abilities are mediated by blindness duration. Our results show that behaviourally, a later onset of blindness is also associated with better performance on a haptic task. Like in the work of Gori et al. [16], our future studies will explore if a shorter period of blindness (i.e., how long the individual has lived without vision) is also associated with different neural correlates for haptic tasks (via EEG).

It is important to consider, however, that the nature of the tasks used to assess haptic processing may account for some of the contradictory findings in the literature. While all tasks rely on haptic perception, they may differ in the degree of cognitive spatial processing they require. Blind participants may outperform sighted individuals in tasks that primarily involve basic haptic discrimination (e.g., identifying matching shapes) where minimal spatial transformation or mental representation is needed. However, when the task requires more complex spatial cognition, such as constructing and manipulating mental spatial representations (as is the case with the task used in our study) blind individuals, particularly those who are early blind, tend to perform worse. Research suggests that the absence of visual experience may impair the ability to generate three-dimensional mental images and may limit the capacity to generate, maintain, and manipulate multiple spatial representations simultaneously [11]. Therefore, we propose that future research should more carefully consider the spatial cognitive demands embedded within haptic tasks. We argue that the task used in our study offers strong ecological validity and more accurately reflects the cognitive demands of real-world haptic interactions.

Our second main finding was that sex differences in haptic processing persisted across both sighted and blind participants, with males outperforming females. This replicates previous results using the same paradigm [1] and aligns with studies showing a male advantage in haptic parallelity tasks [22, 34, 38]. While few studies have examined sex differences in haptic shape or texture discrimination [10, 37], at least one reported superior male performance [10]. To our knowledge, this is the first study to investigate sex differences in haptic ability among blind individuals. One prior study found similar sex differences in a haptic body representation task across both blind and sighted groups [8, 9], where males more accurately judged glove or shoe sizes relative to their own hands or feet. This is consistent with earlier findings in sighted participants [7, 9]. Our current results extend this pattern, suggesting that sex differences in haptic performance, beyond body representation, persist in the absence of visual input. Collectively, these findings indicate that the male advantage in haptic processing is not dependent on visual experience.

To conclude, in the present study we found that visual experience influenced haptic skills. Moreover, the findings from our study suggest that visual experience during development is necessary to calibrate the haptic system. This replicates studies on audition, proprioception, and touch, and therefore supports the hypothesis that vision calibrates spatial representations in the other senses (in this case haptics) during development. Finally, our results showed sex differences in haptic spatial processing that were not influenced by visual experience, suggesting that the male advantage may be independent of visual input.

## Data Availability

Data are available upon reasonable request to the corresponding author.
